# Hydrazine-Assisted Formation of Indium Phosphide (InP)-Based Nanowires and Core-Shell Composites

**DOI:** 10.3390/ma6010085

**Published:** 2012-12-27

**Authors:** Greta R. Patzke, Roman Kontic, Zeinab Shiolashvili, Nino Makhatadze, David Jishiashvili

**Affiliations:** 1 Institute of Inorganic Chemistry, University of Zurich, Winterthurerstr. 190, Zurich CH-8057, Switzerland; E-Mail: roman.kontic@aci.uzh.ch; 2 Institute of Cybernetics, Georgian Technical University, Euli Str. 5, Tbilisi 0186, Georgia; E-Mails: z_shiolashvili@cybernet.ge (Z.S.); n_makhatadze@cybernet.ge (N.M.)

**Keywords:** indium phosphide, nanowires, semiconductors, gas phase deposition, hydrazine, composite materials

## Abstract

Indium phosphide nanowires (InP NWs) are accessible at 440 °C from a novel vapor phase deposition approach from crystalline InP sources in hydrazine atmospheres containing 3 mol % H_2_O. Uniform zinc blende (ZB) InP NWs with diameters around 20 nm and lengths up to several tens of micrometers are preferably deposited on Si substrates. InP particle sizes further increase with the deposition temperature. The straightforward protocol was extended on the one-step formation of new core-shell InP–Ga NWs from mixed InP/Ga source materials. Composite nanocables with diameters below 20 nm and shells of amorphous gallium oxide are obtained at low deposition temperatures around 350 °C. Furthermore, InP/Zn sources afford InP NWs with amorphous Zn/P/O-coatings at slightly higher temperatures (400 °C) from analogous setups. At 450 °C, the smooth outer layer of InP-Zn NWs is transformed into bead-shaped coatings. The novel combinations of the key semiconductor InP with isotropic insulator shell materials open up interesting application perspectives in nanoelectronics.

## 1. Introduction

Among the important group of III–V semiconductor materials [1], indium phosphide (InP) excels through various outstanding properties and finds application in high efficiency photovoltaic cells, integrated circuits and microwave devices operating in the range of 200 GHz, especially for the generation, modulation, detection, transmission and amplification of 1.3 and 1.55 μm wavelength optical signals. InP has an intermediate band gap of 1.34 eV between Si (1.12 eV) and GaAs (1.43 eV), and it combines the highest thermoelectric figure of merit among this compound class together with high electron mobility [2,3,4]. The versatile application potential of InP in forefront technologies, e.g., optoelectronics, spintronics, thermoelectronics and high-speed low-power electronics, as well as in photocatalysis [5], benefits from the formation of InP-based one-dimensional (1D) nanostructures [6]. Single InP nanowire p–n junctions have been identified as promising light emitting diode (LED) materials early on [7]. Recently, for example, periodic two-dimensional (2D) arrays of InP nanowires with embedded InAsP segments were found to be essential for controlling directional light emission in nanowire-based LEDs [8], and self-assembled InP nanowires (NWs) are promising photodetectors [9]. The manifold properties of InP NWs can be further enhanced through doping, e.g., with Zn or main group elements [10], and via core-shell heterostructure formation with other semiconductor materials, such as ZnS [11] or GaP [12]. The majority of these materials have been obtained from MOVPE (metal organic vapor phase epitaxy) or related chemical vapor deposition (CVD) techniques [13,14] proceeding via metal (preferably Au) assisted vapor-liquid-solid (VLS) mechanisms [1,15].

We here present a new hydrazine-assisted and catalyst-free gas phase approach [16] for the formation of InP NWs. High aspect ratio InP NWs are directly obtained from InP precursors, and structure and morphology tuning of InP NWs via the growth parameters is discussed. Furthermore, we demonstrate that the novel synthetic strategy can be extended upon the formation of Zn- and Ga-containing 1D InP core-shell heterostructures.

Whilst the fabrication of 1D nanowire materials with tuned compositions is a challenge in its own right, pristine InP NW growth already gives rise to a fascinating interplay of zinc blende (ZB) and wurtzite (WZ) phases in combination with a variety of twinning phenomena [17]. Although ZB is the preferred structural motif of InP and most other III–V semiconductors, InP NWs often show ZB/WZ polytypism, leading to a staggered type II band alignment in which the ZB conduction band is 129 meV lower than that of WZ [18,19,20]. InP NWs preferably grow perpendicular to the close-packed planes of their respective crystal structures, *i.e.*, in ZB-type InP NWs in [111] and WZ-type InP NWs in [0001] direction [21], which renders alternative growth directions, such as [100] ZB InP nanowires, more difficult to access [22]. The presence of twinning and stacking faults considerably influences the NW properties, as illustrated by photoluminescent blue shifts and unexpectedly large photovoltaic effects through rotational twinning in InP NWs [18,23]. Recently, the room temperature conductivity of InP NWs was furthermore found to depend on the longest ZB segment [18]. InP growth phenomena keep attracting preparative and analytical research interest: Growth patterns of tapered ZB/WZ InP nanowires can be tuned via the SA-MOVPE conditions [17], and a first fully resolved structure of twin planes in InP nanowires was obtained from electron image series reconstruction [24].

Doping processes and heterostructure formation through combination of InP with other III–V elements or compounds are powerful strategies to enhance InP NW applications, as illustrated by single quantum dot nanowire LEDs of InP-InAsP [13] or InP/InAs/InP core-multishell nanowires [14] obtained via VLS/MOVPE processes. Along these lines, axial InP/InSb heterostructures [25] or InP_1−*x*_Sb*_x_* nanowires [26] were furthermore produced from VLS routes.

Despite such rapid progress on InP composites over the past years, there is still room for exploration, as briefly outlined in the following. Combinations of InP with Ga have been reported for Ga/InP NWs [27], and 3D (Ga, In)/GaInP structures were grown on polycrystalline InP substrates [28]. Other reported doping elements for InP comprise Zn [29], Mn [30] and S [13]. However, core-shell composites of InP with gallium oxide are virtually unexplored, whilst Ga_2_O_3_ coatings of GaN [31,32] or ZnO [33] have been fabricated for photocatalytic, sensor and related applications. Given that amorphous Ga_2_O_3_ with a slight excess of Ga displays a fascinating metal-insulator transition with high application potential in once-write storage devices [34,35], its combination with InP would open up an interesting new class of materials. To the best of our knowledge, only the combination of amorphous Ga_2_O_3_ with GaN has been reported to date [36].

Zn P-doping of InP [37] is in the focus of theoretical studies [38,39] and has been applied to tune its photoluminescent properties [40], as well as the spacing of twinning superlattices [41]. Moreover, InGaAs/InP transistor performance benefits from Zn doping [42,43]. Zn-based shell materials for InP NW heterostructures, however, are far less explored, and their combination with oxidic Zn-coatings has not been reported to the best of our knowledge. Few examples, such as TCO ZnO films deposited on InP substrates, still outline the potential of such materials [44].

In the following, we introduce a hydrazine-based direct vapor-solid route to InP nanowires with different structural and twinning motifs. Next, we illustrate the application of this approach upon the all-in-one growth of InP NWs with Ga- and Zn-containing shells. Morphological varieties of Zn-based core-shell InP heterostructures are investigated in detail, and the influence of additional source elements on the InP deposition process is discussed.

## 2. Results and Discussion

### 2.1. Hydrazine-Assisted Gas Phase Formation of InP Nanowires

#### 2.1.1. Experimental Parameters

The pyrolytic decomposition of hydrazine in the presence of 3 mol % H_2_O leads to a variety of highly reactive molecular species that promote the decomposition of the InP source and of gas phase transport processes towards deposition of InP nanowires. As illustrated in previous studies on the formation of phase pure germanium nitride nanowires from a related hydrazine/water atmosphere [16], semiconductor formation is favorable over the oxide formation pathway. The hydrazine-assisted deposition route establishes an elegant one-step and catalyst-free conversion of bulk InP sources into high aspect ratio InP nanowires (NWs) via vapor-solid mechanisms, as discussed in the following.

Sublimation of InP source material in the hydrazine atmosphere of the evacuated quartz reactor provides phosphorus and indium species for the formation of InP nanowires on glass and Si substrates. Phosphorus species can emerge from two processes: First, the saturation pressure of P is *ca.* 10^−6^ atm at the average preparation temperature of 615 °C, thus providing sufficient quantities for NW growth [45]. Furthermore, atomic hydrogen generated via hydrazine decomposition has been reported to reduce the InP decomposition temperature by 230 °C, thereby promoting the formation of P_2_, P_3_ and PH_3_ species [46]. Furthermore, indium is formed through decomposition of InP:

(1) $$\text{InP}\left(\text{s}\right)\;\overset{\;}{\to }\;\text{In}\left(\text{s}\right)+0.5\;{\text{P}}_{2}(\text{g})$$

Indium transfer to the substrate surface is probably mediated through the formation of volatile In_2_O molecules emerging from the small quantities of added H_2_O and In. However, further studies in their own right are required to clarify the complex reaction equilibria involved in InP NW deposition.

**Figure 1:**
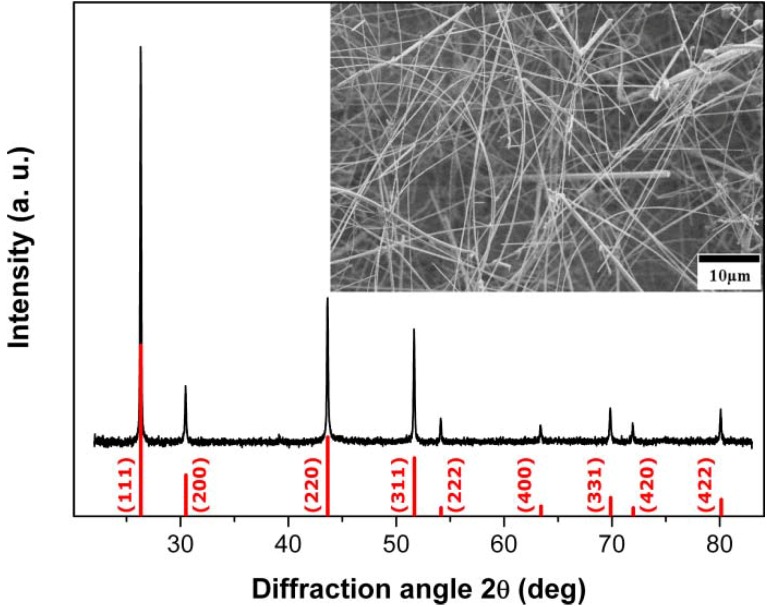
Representative X-ray diffraction (XRD) pattern and scanning electron microscope (SEM) image (inset) of zinc blende (ZB)-type indium phosphide (InP) wires deposited on a Si substrate at 440 °C (reference pattern: ICSD 73-1983).

Concerning the reaction parameters, substrate material and deposition temperature were found to exert a key influence on the phase purity and morphology of the obtained InP products. Glass substrates led to the formation of a mixture of InP and In with no special morphologies, whilst Si substrates afford phase pure InP nanowires with zinc blende (ZB) structure (Figure 1). Therefore, the following discussion mainly refers to InP NWs grown on Si substrates.

#### 2.1.2. Temperature-Morphology Relations among InP NWs

The substrate temperature significantly influences the morphology and crystallinity of the obtained InP materials, which can be differentiated into products obtained at higher temperatures (up to 540 °C on glass substrates) and NWs grown at 440 °C. Products grown at a substrate temperature of 540 °C mostly display microscale dimensions with a lower degree of anisotropy and characteristic spherical tips that are indicative of a VLS growth mechanism (Figure S1). High aspect ratio NWs with characteristic zigzag shapes are obtained in smaller quantities and will be discussed further below.

**Figure 2:**
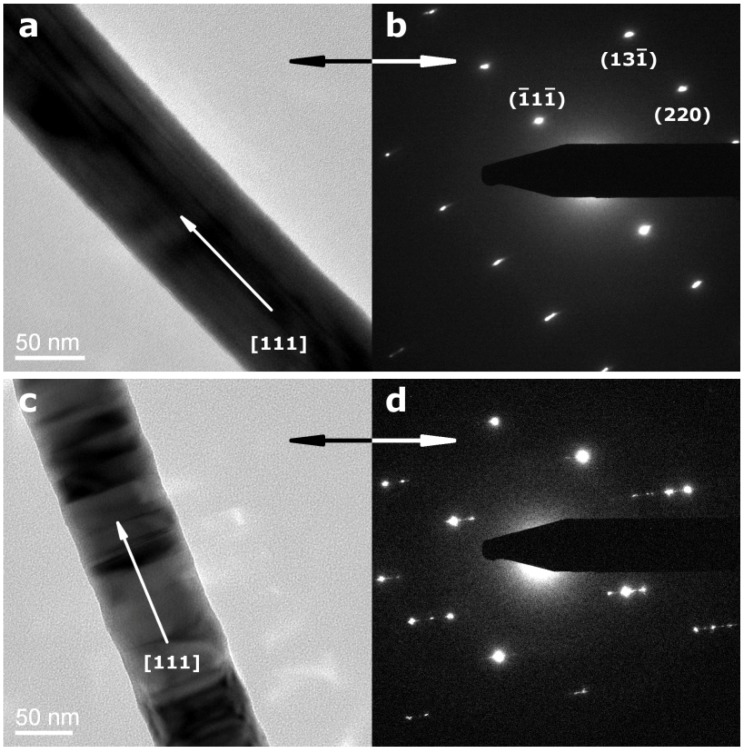
Transmission electron microscopy (TEM) images and selected area electron defraction (SAED) patterns of ZB-type InP nanowires grown at 440 °C (**a**,**b**) 92 nm diameter InP NWs with less defects and corresponding SAED pattern; (**c**,**d**) randomly twinned InP NWs with average diameter of 90 nm and SAED pattern of one of such nanowires, which displays the striation of diffraction spots caused by the introduction of random twins.

**Figure 3:**
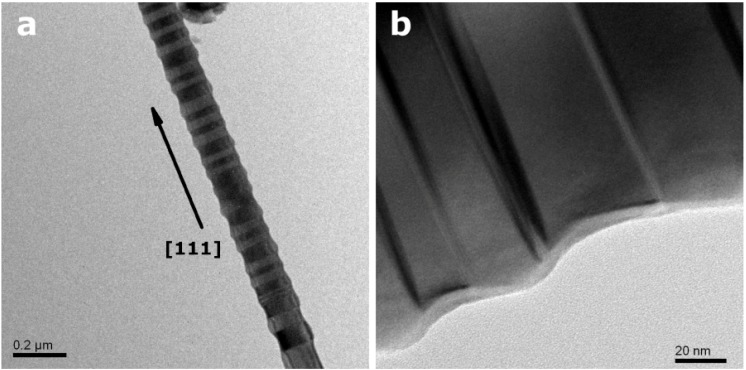
TEM image of more regularly twinned ZB-type InP NWs grown along [111].

InP NWs grown at 440 °C on Si substrates display high aspect ratios with minimum diameters <40 nm and lengths >80 µm (inset in Figure 1). The absence of droplet particles at the tips of NWs grown at lower temperatures supports a genuine vapor-solid (VS) growth mechanism. If the same mechanism would be at work at higher and lower temperatures, residual droplets from quenching processes would have been observed after InP deposition at 440 °C as well. However, their presence at 540 °C indicates a VLS mechanism (Figure S1), which is changed into VS growth at 440 °C without droplet formation. Furthermore, the absence of tapering (inset in Figure 1) excludes side-wall diffusion or deposition processes for NWs grown at 440 °C and lower temperatures. TEM investigations of the InP NWs deposited at 440 °C reveal that they can be differentiated into two NW types (Figure 2). Type 1 NWs (Figure 2a,b) display the characteristic [111] growth direction of ZB-type InP wires, and the observed lattice spacing of d_111_ = 0.340 nm, calculated from the SAED pattern in Figure 2, agrees well with the d-spacing for this plane in ZB InP. Although asymmetric reflections in the SAED patterns indicate a slight structural distortion (Figure 2b), the NWs display uniform diameters, straight walls and little indication of strain or disorder (Figure 2a). Type 2 ZB InP NWs grown along [111] (Figure 2b,c), however, display significant rotational twinning, which can be explained in terms of the low stacking fault energy of the (111) planes in InP [47]. Random twinning is indicated by an irregular sequence of different domains in the TEM image (Figure 2c) and striation of diffraction spots in the SAED (Figure 2d). The stacking order is changed through insertion of twin planes, which create small WZ segments between two ZB domains. The resulting directional change of 39° leads to the irregularly shaped NW walls. Figure 3 finally shows a third type of ZB InP NWs with a more regular rotational twinning pattern, as shown in preceding reports [48].

Further structural and elemental analyses on the InP nanowires were conducted with high-angle annular dark field scanning transmission electron microscopy (HAADF-STEM). Z contrast effects clearly indicate that the type 1 InP NWs are indeed uniform in composition and structure, whilst type 2 NWs display characteristic segmentation due to twinning and WZ segment insertion (Figure S2). EDX analyses on selected spots (Figure S2) are in line with stoichiometric InP. Most importantly, the formation of oxide and nitride side products can be excluded. The deposition temperature of 440 °C is probably too low to permit InN growth, and oxide side products are likely to be reduced *in situ* through the generated hydrogen species.

#### 2.1.3. Zigzag Shaped InP Nanowires

**Figure 4:**
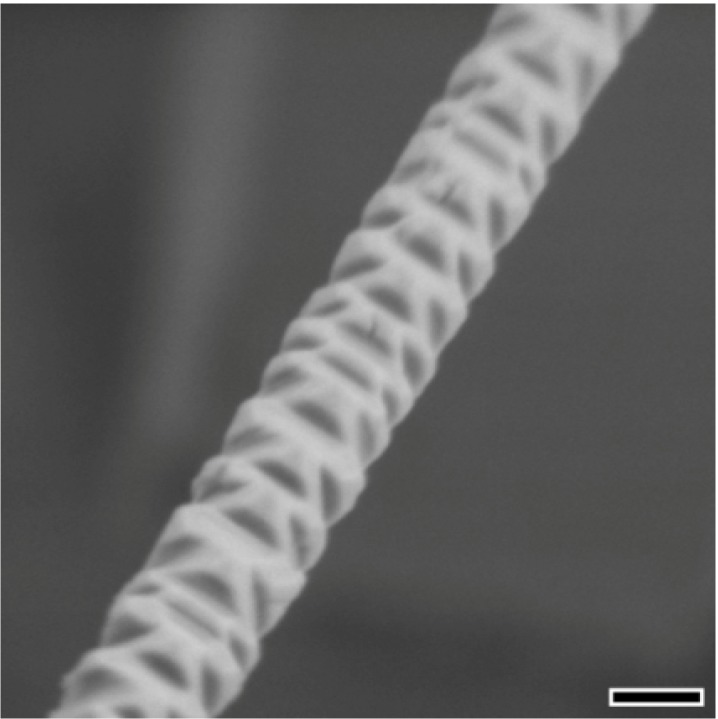
InP nanowire with edged surfaces grown at 540 °C on a glass substrate (scale bar = 200 nm).

Figure 4 shows a representative example of ZB-type InP NWs with a characteristic zigzag growth pattern obtained at 540 °C. This morphology attracted considerable interest for device applications due to unique structural and optical properties, e.g., shifts of PL emissions [49,50]. However, preceding routes to such zigzag NWs require the presence of ZnSe as a promoter and higher formation temperatures of 1200 °C in quartz tubes. Interestingly, our experimental setup is capable of generating these interesting NW types in the absence of promoters at considerably lower temperatures. Possible driving forces for their formation are induction through hydrazine decomposition products or intense twinning in combination with a high growth rate.

### 2.2. Mixed Nanowire Growth from InP–Ga Sources

#### 2.2.1. Scope and Synthesis

**Figure 5:**
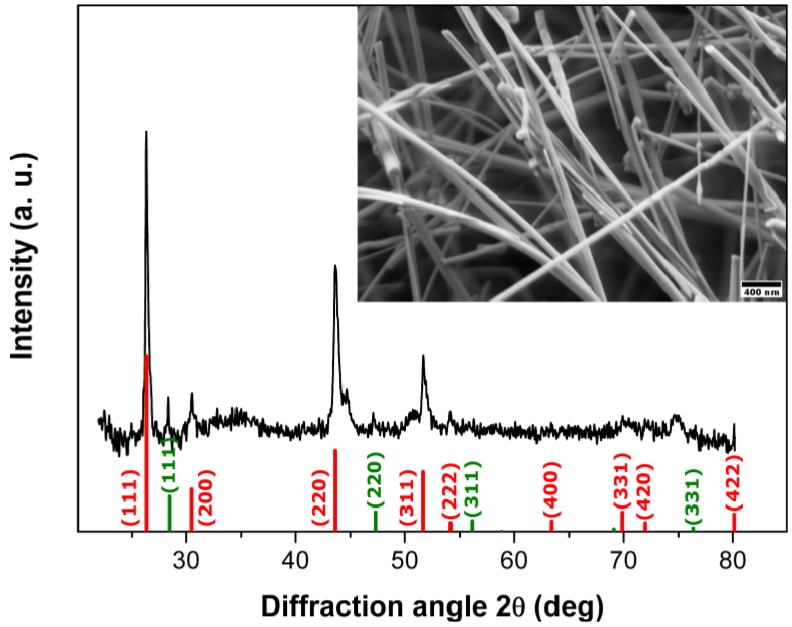
Representative powder X-ray diffraction (PXRD) pattern of ZB InP:Ga nanowires (NWs) (red = reference pattern: Inorganic Crystal Structure Database (ICSD) 73-1983; green = Si from substrate) with SEM image of NWs grown at 350 °C (inset).

As outlined above, the coating of InP nanowires with several types of Ga-containing shells is both of application-oriented and structural research interest. Therefore, the protocol for InP NW growth was modified with respect to the source material. A small piece of pure Ga was introduced into the reactor together with a 400 µm thick single crystalline InP platelet at an approximate InP:Ga surface ratio of 30:1. High aspect ratio NWs are formed from InP–Ga sources in the temperature range between 350 and 425 °C, and neither tapering nor catalyst droplets are observed among the products. Higher deposition temperatures, however, led to less regular wire shapes due to higher growth rates, accompanied by a higher tendency towards stacking faults and twinning. Figure 5 shows a representative PXRD pattern and SEM image of ZB InP–Ga NWs grown at 350 °C with circular cross sections, lengths up to several tens of micrometers and diameters in the range of 50–100 nm. Other than pristine InP NWs with frequently uneven edges due to rotational twinning, the majority of the InP–Ga NWs display smooth edges, regardless of twinning (Figure S3).

#### 2.2.2. Structural Properties

**Figure 6:**
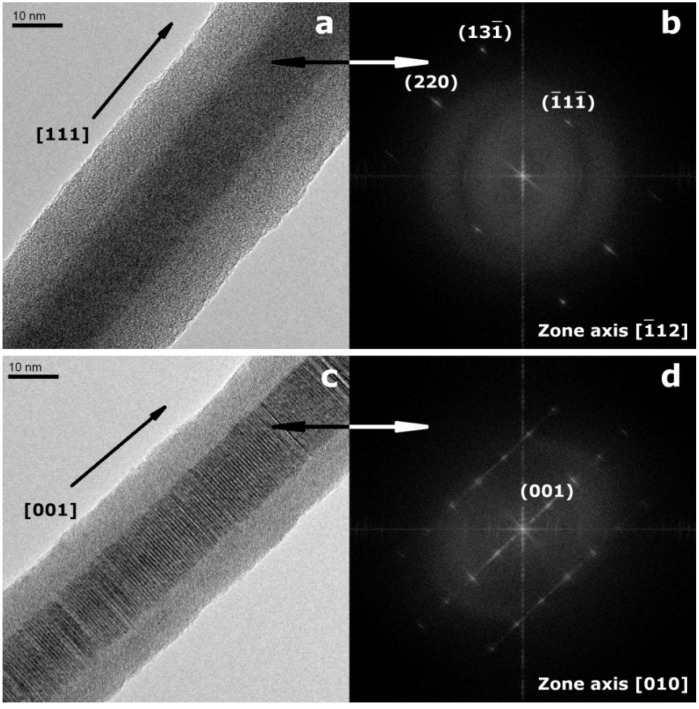
Representative TEM image of (**a**) non-twinned ZB-type InP–Ga NW with average core diameter of 14 nm and average shell thickness of 11 nm; (**b**) corresponding Fast Fourier Transform (FFT); (**c**) TEM image of twinned ZB-type InP–Ga NW with average core diameter of 14 nm and average shell thickness of 8 nm; (**d**) corresponding FFT.

TEM investigations of InP–Ga NWs display a core-shell structure with smooth edges and crystalline cores with diameters smaller than 15 nm, *i.e.*, below the exciton Bohr radius in InP (r_B_ = 15 nm) [51], thus indicating that the properties of the material are influenced by quantum confinement effects. The addition of Ga to the source material exclusively promotes the formation of a certain extent of InP–Ga NWs with WZ-type crystalline InP cores [15] (Figure 6c,d) in addition to common ZB-type core (Figure 6a,b) that was obtained from all other setups in the present study. Figure 6 illustrates typical growth directions along [111] for the ZB-type and [001] for the WZ-type InP core, respectively.

All observed d-spacings for ZB-type InP nanowires fall within the literature data ranges, and they do not point to a lattice parameter decrease, which would be expected in case of a significant Ga incorporation [52]. Interestingly, the NW cores appear to be mostly free of Ga despite its saturation vapor pressure of *ca.* 10^−8^ Torr at the preparation temperature of 400 °C in combination with facile sublimation via volatile Ga_2_O molecule formation in the presence of hydrogen and water vapor. These results are backed by HAADF-STEM analyses on NWs with average diameters around 40 nm in combination with EDX spot measurements (Figure S4). The NW shells consist mainly of Ga and O, whilst the NW cores display massively stronger InP signals and constantly low Ga/O ratio. All in all, these results indicate the formation of crystalline InP NWs with an amorphous gallium oxide shell. Given the interesting metal-insulator transition of amorphous Ga_2_O_3_ [34], this new type of NW composites opens up exciting options for theoretical studies. Additionally, the isotropic electrical characteristics of the amorphous Ga_2_O_3_ in the core-shell InP–Ga_2_O_3_ composite NWs render them interesting candidates for the fabrication of wrap-gate metal insulator semiconductor transistors.

The complex mechanism of subsequent InP and gallium oxide deposition to yield core-shell nanowires is currently under investigation. The absence of Ga doping in the NW core suggests that gallium suboxide molecules are segregated from the growing InP nanowires, followed by their oxidation to Ga_2_O_3_ at the nanowire edges. The rather mild formation conditions are probably insufficient to induce recrystallization of gallium oxide, thereby leading to an amorphous gallium oxide coating of the InP nanowires.

### 2.3. Hydrazine-Assisted Nanowire Growth from InP–Zn Source Materials

#### 2.3.1. Scope and Synthesis

As Zn-containing shells for InP nanowires are far less explored than bulk doping with Zn [40,41], the above procedure for the synthesis of InP–Ga NWs was modified through replacement of Ga with Zn in the source materials. In the following, we demonstrate that the presence of Zn induces different growth patterns and morphologies of the NW products. Structure and shape of InP–Zn NWs are compared to pristine InP, as well as to InP–Ga NWs to demonstrate the flexibility of the hydrazine-assisted route.

#### 2.3.2. Influence of the Reaction Temperature

**Figure 7:**
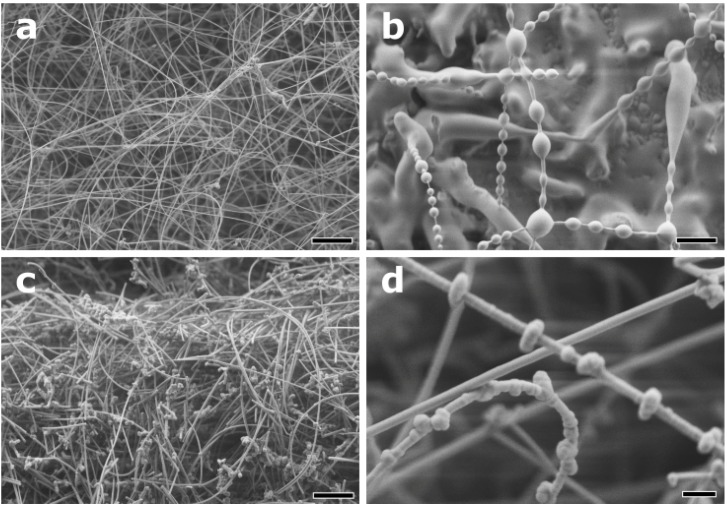
Representative SEM images of nanowires grown from InP/Zn sources at (**a**) 400 °C; (**b**) 420 °C; (**c**,**d**) 650 °C (scale bars = 2 μm for (**a**–**c**) and 400 nm for (**d)**).

**Figure 8:**
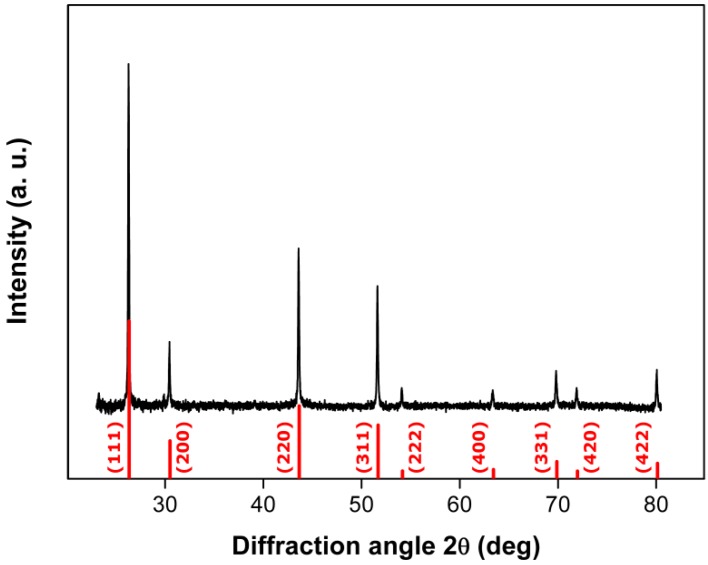
PXRD pattern of high aspect ratio ZB-type NWs grown at 400 °C from InP/Zn sources (reference pattern: ICSD 73-1983).

In analogy to the above-mentioned growth of InP nanowires, products obtained from InP–Zn sources display a tendency towards less regular morphologies at higher temperatures (Figure 7). Uniform and high aspect ratio ZB-type NWs are obtained at 400 °C (Figure 7a and Figure 8), and higher deposition temperatures favor the formation of NWs covered with bead-like particles in a rather regular fashion (Figure 7b). The alignment of these bead particles becomes more random with coarsened particle shapes at 650 °C (Figure 7c,d).

**Figure 9:**
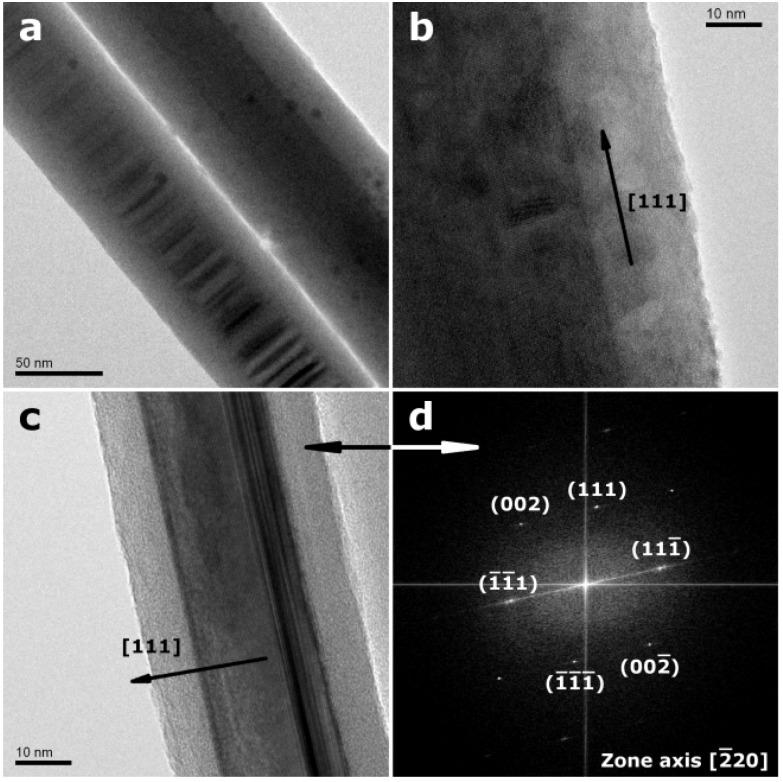
Representative TEM analyses of ZB nanowires grown from InP/Zn sources at 400 °C: (**a**,**b**) grown along [111]; (**c**) grown perpendicular to [111] and (**d**) Fast Fourier Transform of (**c**).

In the following, InP–Zn derived NWs grown at 400 °C are subjected to a more detailed TEM analysis. Representative TEM images in Figure 9 clearly show the presence of core-shell particles with ZB cores and amorphous shells that bear resemblance to the aforementioned InP–Gallium oxide composites (Figure 6). Interestingly, however, the presence of Zn in the source material leads to a wider variety of growth directions compared to the pristine InP and InP–Ga systems. Whilst Figure 9a,b shows twinned and untwinned ZB InP rods growing along the expected [111] direction, Figure 9c illustrates the presence of alternative growth directions, e.g., perpendicular to [111] with the characteristic (111) stacking faults running along the NW axis.

HAADF-STEM analyses coupled with EDX methods (Figure S5) of Zn-containing InP NWs show that Zn is located in the amorphous NW shell region together with significant amounts of oxygen and phosphorus. Other than in the case of NWs grown from InP–Ga sources, InP–Zn based composites are coated with a complex material, probably zinc phosphate. Further investigations on the phase assignment are in progress.

Bead-decorated composite NWs grown at 420 °C bear close resemblance to InP NWs coated with pearl-shaped ZnS particles [11]. Whereas the morphologically related InP–ZnS NWs were obtained from InP and ZnS powders at rather high temperature (1250 °C) in preceding studies [11], we lowered the deposition temperature for comparable nano-architectures to 420 °C with no need for sulfur as an additional precursor element.

**Figure 10:**
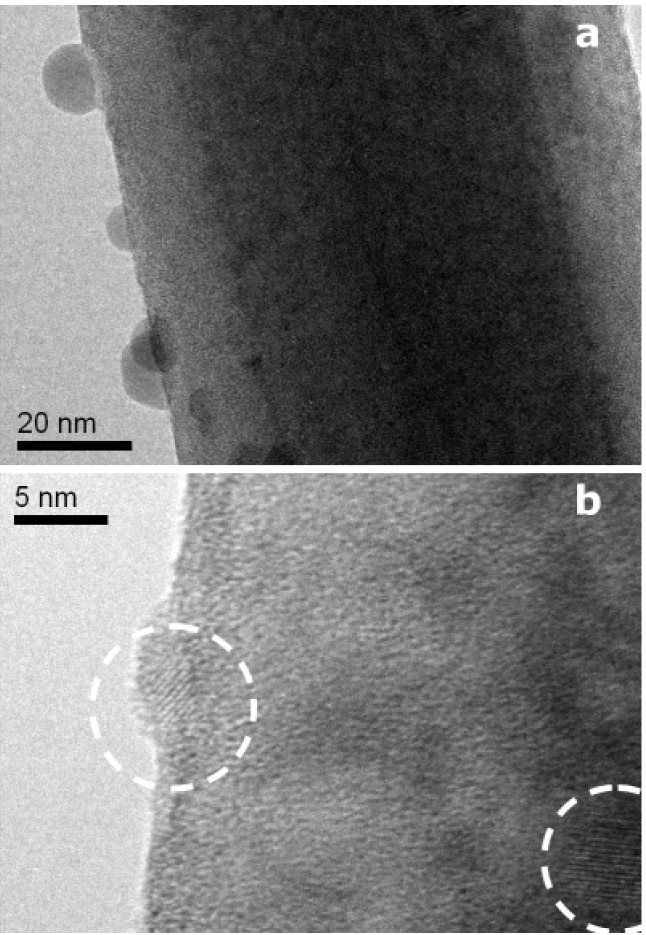
InP:Zn nanowires covered at 400 °C with (**a**) amorphous droplets and (**b**) crystalline droplets of a Zn-rich shell material.

The formation of bead-coated InP NWs already sets in to a lower extent at 400 °C, and it is fully developed at 450 °C (Figure 10). Elevated temperatures might favor shell shrinkage as a result of surface tension, and the resulting local crystallization processes of the NW shell are indicated by the presence of partially crystallized surface beads (Figure 10b). Crystallization at such low temperatures may be enabled by quantum size effects, and elevated temperatures (650 °C, Figure 7d) are likely to induce Ostwald ripening towards formation of larger surface particles. These complex morphology and crystallinity transformations on the NW surface are subject to follow-up studies.

## 3. Experimental Section

### 3.1. Sample Preparation

Commercial grade hydrazine solution in water (50%, former Soviet standard N5832-65) was distilled at 113 °C with an Ar flow (100–150 sccm/min) during the first 20 min. The water content after distillation was determined as 3 mol % using a IRF-22 refractometer; this remaining water content plays an important role during the synthesis, as discussed below. The reaction was conducted in a cylindrical quartz reactor, which was connected to a gas/vacuum system and had a resistive heater attached to its flat bottom.

**Figure 11:**
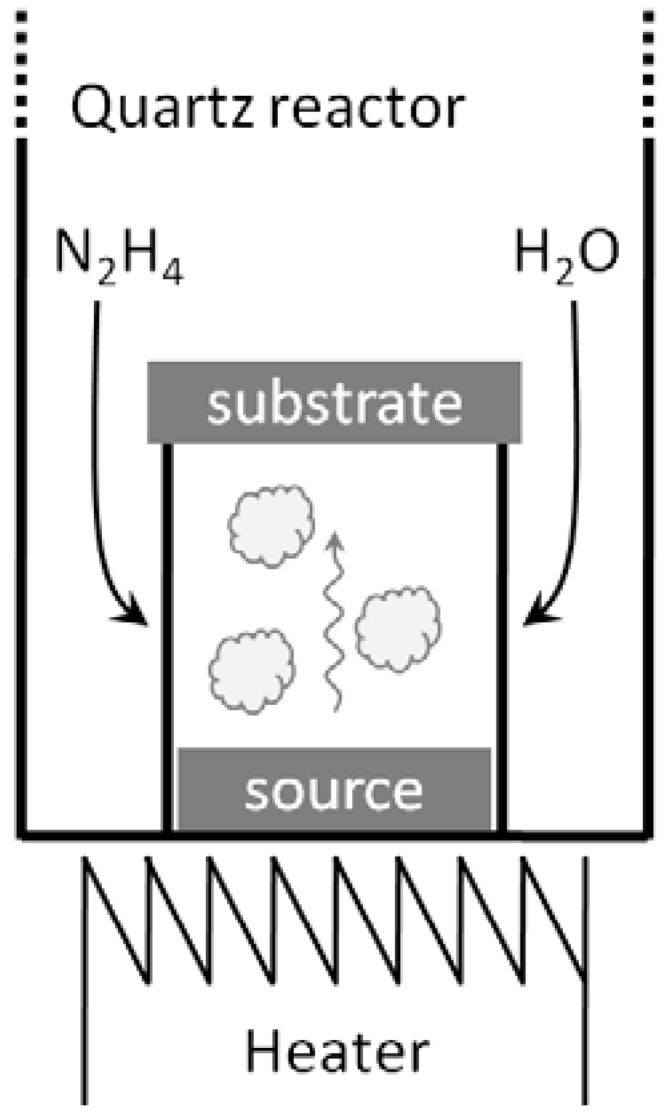
Cylindrical quartz reactor for nanowire growth.

The source InP wafer (FIET-1 type Te doped (100) oriented single crystalline n-InP with carrier concentration of 5.8 × 10^16^ sm^−3^) was placed at the bottom of the reactor, and the substrate (silicon wafer or glass) was placed above it, separated by a quartz spacer (Figure 11). The reactor was first evacuated and then filled with the saturated pressure of the hydrazine vapor (10 Torr). The temperature of the source was set by the resistive heater. The substrate was subsequently heated due to radiative and convective heating from below, and its temperature was determined by the source temperature and the distance from the source.

### 3.2. Characterization

Samples were analyzed by scanning electron microscopy (SEM) using a LEO 1530 (FEG) microscope with 1.8 keV electrons. Transmission electron microscopy (TEM) images were recorded on a Philips CM12 operated at 100 keV with a W cathode and a FEI Tecnai F30 (FEG) equipped with an energy dispersive X-ray (EDX) and a high-angle annular dark-field (HAADF) detector; samples were ultrasonicated in ethanol prior to deposition on carbon coated copper grids. X-ray powder diffraction analyses were conducted on a STOE STADI-P2 diffractometer in transmission mode (flat sample holders, Ge monochromated CuK_α1_ radiation) equipped with a position-sensitive detector. Samples were fixed with Mylar foil and Wacker P4 silicon glue.

## 4. Conclusions

We established a new one-step vapor-solid approach to high aspect ratio InP NWs via chemical transport of crystalline InP sources in hydrazine (3 mol % H_2_O) atmospheres. The process affords ZB-type InP NWs with uniform diameters around 20 nm and lengths up to several tens of nanometers at 440 °C. The clear-cut particle morphologies point to a direct vapor-solid growth mechanism. Reaction temperature is the key parameter for pristine InP nanowire formation, and the particle size increases towards higher deposition temperatures. Furthermore, this flexible preparative route brings forward core-shell InP nanowires from mixed precursor materials. InP–Ga sources afford the first example of InP NWs coated with amorphous gallium oxide shells. Nanocables consisting of ZB InP cores with diameters <20 nm covered with a thin gallium oxide layer are formed at low deposition temperatures around 350 °C. Likewise, InP–Zn sources generate special core-shell NW architectures via one-pot and low-temperature hydrazine routes. The process can be steered towards formation of smooth InP NWs with amorphous Zn/P/O-shells at 400 °C. They gradually transform into a new type of bead-coated InP:Zn-NWs under mild deposition conditions around 450 °C. These NW composites consist of a crystalline InP core covered by an isotropic amorphous insulator shell, thus opening up new application perspectives in nanoelectronics. Furthermore, the observed morphology transformation processes serve as a starting point for theoretical and crystallographic studies, especially concerning the unprecedented combination of InP with amorphous gallium oxide as a versatile conductor.

## Supplementary Materials

Supplementary File 1
