# Supplementary material for: Hydrazine-Assisted Formation of Indium Phosphide (InP)-Based Nanowires and Core-Shell Composites
Source: Materials (Basel). 2012 Dec 27;6(1):85–100. doi: 10.3390/ma6010085 (PMC5452112; doi:10.3390/ma6010085)
Supplement: Supplementary File 1 [file materials-06-00085-s001.pdf]

Materials **2013**, *6*, 1-x manuscripts; doi:10.3390/ma60x000x

OPEN ACCESS

materials

ISSN 1996-1944

www.mdpi.com/journal/materials

Supporting Information

Figure S1. Representative scanning electron microscope (SEM) image of products grown from an InP source at 540 °C on glass substrate (scale bar = 2 μm).

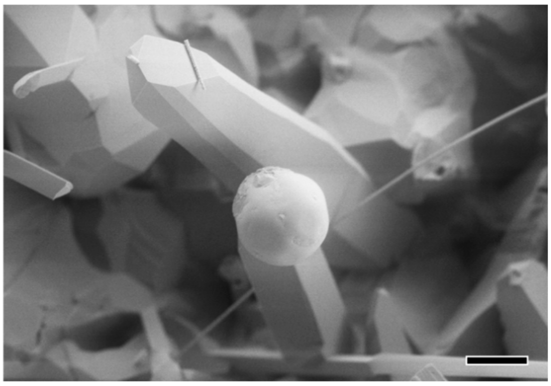

Figure S2. High-angle annular dark field scanning transmission electron microscopy (HAAD-STEM) image and energy dispersive X-ray (EDX) analyses of InP wires grown on Si substrate at 440°C (C and Cu signals arise from the Transmission electron microscopy (TEM) grid and partial surface contamination due to air exposure).

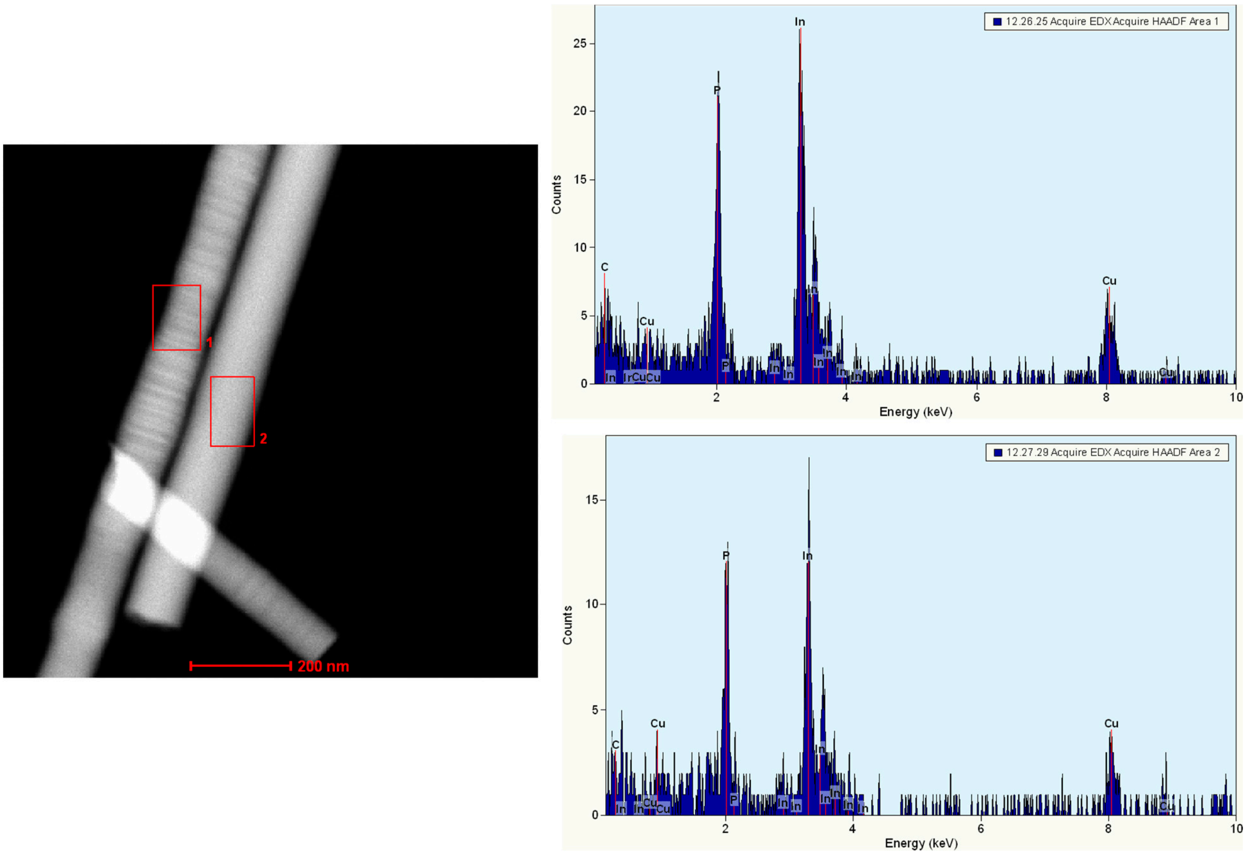

Figure S3. Representative TEM images of InP–Ga nanowires.

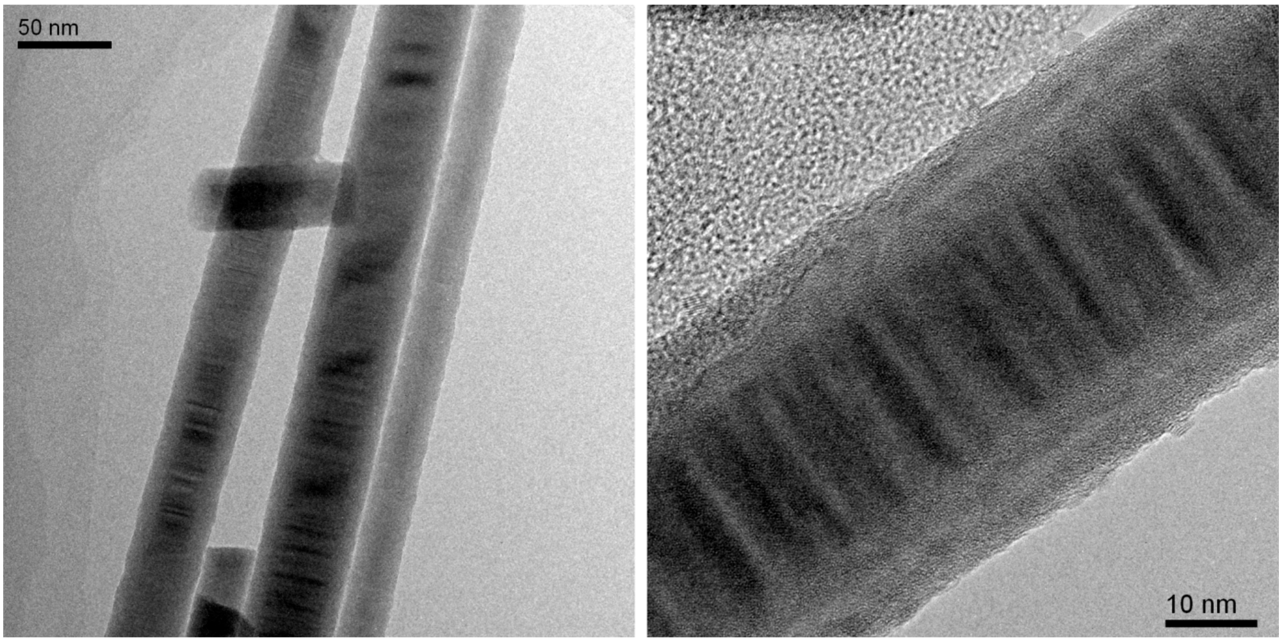

Figure S4. Scanning transmission electron microscopy energy dispersive X-ray (STEM-EDX) analyses of InP-Ga nanowires (grown on Si substrate at 350 °C).

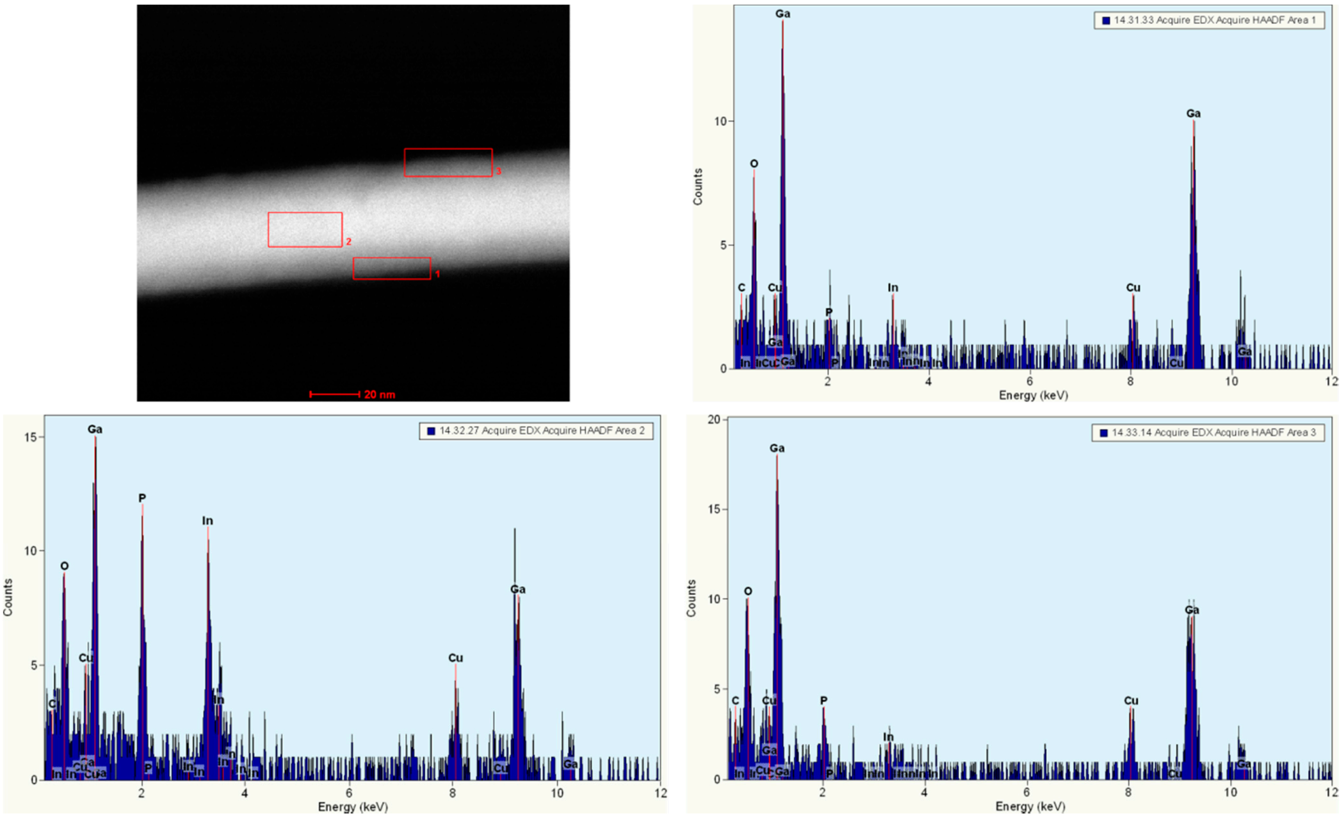

Figure S5. STEM-EDX analyses of InP-Zn wires (grown on Si substrate at 400° C).

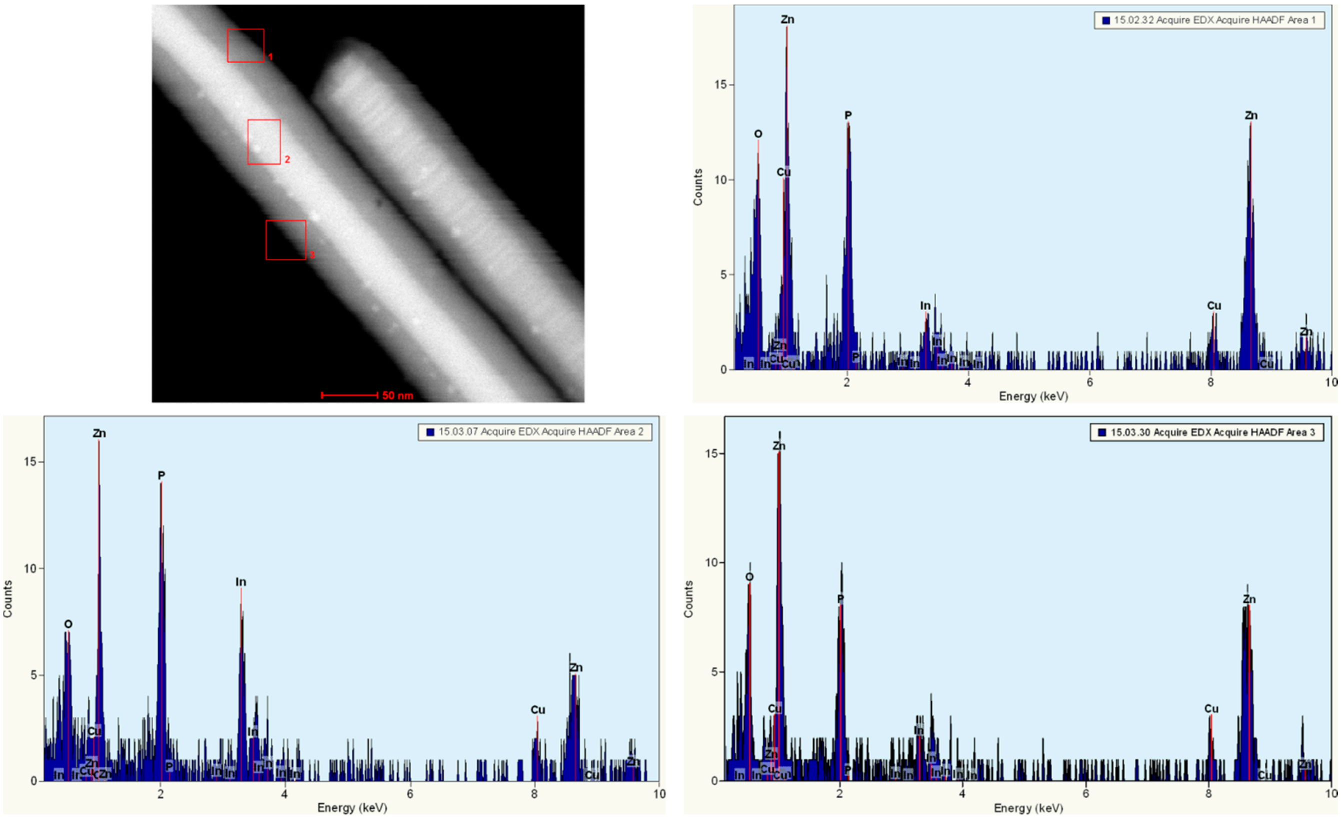

© 2013 by the authors; licensee MDPI, Basel, Switzerland. This article is an open access article distributed under the terms and conditions of the Creative Commons Attribution license (http://creativecommons.org/licenses/by/3.0/).
